# Twenty years of *in vitro* nanotoxicology: how AI could make the difference

**DOI:** 10.3389/ftox.2024.1470439

**Published:** 2024-09-18

**Authors:** Luisa Campagnolo, Valentina Lacconi, Joanna Filippi, Eugenio Martinelli

**Affiliations:** ^1^ Department of Biomedicine and Prevention, University of Rome Tor Vergata, Rome, Italy; ^2^ Department of Electronic Engineering, University of Rome Tor Vergata, Rome, Italy

**Keywords:** *in vitro* nantoxicology, artificial intelligence, *in vitro* models, machine learning, engineered nanomaterials (ENM)

## Abstract

More than two decades ago, the advent of Nanotechnology has marked the onset of a new and critical field in science and technology, highlighting the importance of multidisciplinary approaches to assess and model the potential human hazard of newly developed advanced materials in the nanoscale, the nanomaterials (NMs). Nanotechnology is, by definition, a multidisciplinary field, that integrates knowledge and techniques from physics, chemistry, biology, materials science, and engineering to manipulate matter at the nanoscale, defined as anything comprised between 1 and 100 nm. The emergence of nanotechnology has undoubtedly led to significant innovations in many fields, from medical diagnostics and targeted drug delivery systems to advanced materials and energy solutions. However, the unique properties of nanomaterials, such as the increased surface to volume ratio, which provides increased reactivity and hence the ability to penetrate biological barriers, have been also considered as potential risk factors for unforeseen toxicological effects, stimulating the scientific community to investigate to which extent this new field of applications could pose a risk to human health and the environment.

## The last 20 years of nanotoxicology

Pioneering *in vitro* studies have focused on understanding the basic interactions between nanomaterials and biological systems and the mechanisms of nanomaterial cellular uptake. Moreover, in the frame of risk assessment, assays have been developed to evaluate cytotoxicity, genotoxicity and oxidative stress, leading to the production of high-volume data on the mechanisms through which nanomaterials may exert adverse health effects. The advent of the OMICS era has increased the data volume even more. From 2D models to 3D models, increasing complexity has been added to the developed models, with the aim to more faithfully *in vitro* reproduce tissues and organ systems. Starting from simple 2D cell cultures, generally consisting of tumour cell lines, we have assisted to the development of more sophisticated models using primary cells, often combined in complex cultures to create new approach methodologies (NAMs) as tools for a more reliable safety assessment of nanomaterials. Nearly all tissues have been modeled *in vitro*, including the respiratory, immune, nervous, digestive, and reproductive systems. Moreover, primary and internal biological barriers have been reproduced using advanced *in vitro* models to represent the skin, lung and gut barriers, as well as the blood-placental and blood-brain barriers. A wide variety of nanomaterials have been tested for their ability to affect barrier integrity and permeability, as well as for their effects on the tissues across the barriers (Aengenheister et al., 2019; Boos et al., 2021; Lacconi et al., 2022).

Additional complexity has been added by the development of organoids and organ-on-chip models, for which, on the other hand, the use of NM has demonstrated beneficial in reproducing with better properties the organ of interest (Boos et al., 2021; Shen et al., 2023).

Indeed, organoids have been used as physiologically relevant models of the brain, colon, and lung to test the toxicity of different NMs, such as, among others, carbon-based, silver, and silica nanoparticles (Issa et al., 2024; Jiang et al., 2020; Kastlmeier et al., 2022; Park et al., 2020). They have been also used to investigate nano-bio interactions for nanomedicine applications (Bao et al., 2023) and to improve organoid formation (Shen et al., 2023). Organ-on-chip technology is still in its infancy (Rogal et al., 2022); however, it presents several invaluable advantages, such as reproducing human organ physiology and tissue-tissue interactions in minimal dimensions and providing human-relevant data (Bendre et al., 2022). In this respect, organ-on-chip models of different organ systems, including skin, lung, liver and kidney and the blood-placental-barrier have been developed for hazard identification and risk assessment of NMs (Kohl et al., 2021; Yin et al., 2019).

The increase in model complexity, although determining a greater model validity, raises a major problem in term of experimental standardization and data interpretation and management.

Standardization represents a critical point to allow experimental reproducibility and interpretation and potential applications of the results. However, the interpretation of results has often been challenging, as many variables may affect the data, including the use of unrealistic concentrations, which may demonstrate toxicity for otherwise low-toxic nanomaterials. Indeed, the raising attitude to report sensational results, soliciting the fear from the community, has in some cases led to the perception of nanotechnology as a potential serious harm for human health, overshadowing its undoubted benefits. In this respect, biosafety assessment has become mandatory for the development of any new nanomaterial, and over the last two decades, nanotoxicology studies have allowed the identification of the physicochemical properties driving toxicity and the emergence of the concept of safe and sustainable by design (SSbD) as the basis for the development of new materials.

A number of efforts have been made to develop strategies for reducing the testing of NMs while characterizing their potential risks to human health and the environment. The concepts of quantitative structure-activity relationship (QSAR), grouping and read across have been revisited for their application to NMs. QSAR is based on computerized approaches to correlate toxicity and chemical structure and provide information for grouping and read-across. These strategies represent the first attempt to apply modelling and artificial intelligence algorithms to identify and classify NM toxicity. In the frame of grouping, over the last years, a number of physico-chemical parameters have been tentatively recognized as important to characterise NM and their potential toxicity, including among others, chemical identity, size and particle size distribution, aspect ratio, surface activity and reactivity and aging, and strategies have been designed to identify the critical criteria for NM grouping (Lynch et al., 2014). Nevertheless, the lack of detailed descriptions of protocols and standardization in data collection have hampered the possibility to develop defined algorithms for unbiased grouping of NMs, although grouping would greatly reduce the costs for NM risk assessment and would improve the SSbD development of new NMs (Drasler et al., 2017; Ribeiro et al., 2017). Moreover, in terms of costs, grouping, by allowing the categorization of NMs into defined categories, represents the first step to allow the application of read-across strategies to fill data gaps for NMs of poorly characterized hazard, and to position them in groups of NMs with well characterized nano-bio interaction. The application of read-across approaches, however, requires the identification of a wide range of physicochemical parameters that precisely characterize the NM, limiting their use for some NMs. Moreover, the application of read-across is strictly related to the endpoint of interest, making even more complex its use. The European Chemical Agency (ECHA) has recently produced a guidance document, Read-Across Assessment Framework (RAAF), as a tool for the assessment of grouping and read-across according to the REACH regulations. This document provides valuable information for its application also to NMs (ECHA, 2017).

## AI application to nanotoxicology, how far are we?

Although mathematical modelling has provided significant progress in the management of the large volume of data generated by nanotoxicology studies, challenges remain, including the complexity of data interpretation, variability in experimental results, and the necessity for high-throughput analysis. Moreover, the correlation between data and information is often not trivial to identify since, as already discussed, the quality of data may not always allow the extraction of information. A solution to these challenges may be represented by Artificial Intelligence (AI), defined as “a machine-based system that can, for a given set of human-defined objectives, make predictions, recommendations or decisions influencing real or virtual environments”(OECD AI, 2020). Algorithms of AI may be used to manage and integrate a vast amount of data produced in the context of nanotoxicology and hence provide information for the identification of shared features for grouping and read-across. These algorithms could easily manage, for example, the high-volume data obtained by OMICS approaches, whose quality generally represents the optimal information to feed machine learning. However, despite their high accuracy, AI model predictions are often perceived as black boxes, especially in healthcare decision-making. Therefore, the explainability or interpretability of AI model predictions is crucial for providing comprehensible cause-effect relationships (Holzinger et al., 2019). In recent years, significant attention has been devoted to developing various models, such as Causal and Graph Neural Networks, which can compute the causal effect of each feature on the output (Chattopadhyay et al., 2019; Simon et al., 2024). However, the application of AI to nanotoxicology is still in its infancy. To date, machine learning approaches have been applied to silver nanoparticles with different sizes and surface coatings to predict protein corona formation, a parameter that may greatly influence nano-bio interactions. A similar approach has been used to identify the parameters mediating silver nanoparticle cytotoxicity by extracting data from 162 independent experiments, demonstrating exposure concentration and duration, zeta potential, particle size, and coating as the most relevant features. Similar approaches have been applied to extract predictors of cytotoxicity for other types of NMs (Martin et al., 2023; Regonia et al., 2022). In this respect, recently, we reported the use of machine learning algorithms to identify nanoparticle chemical properties and experimental settings relevant for predicting cytotoxicity of 12 different NMs (Conti et al., 2022). For this study, we used a multicentre database of data collected under the EU funded COST Action MODENA (MODENA, 2012; https://www.cost.eu/actions/TD1204/). A regressor model based on extreme gradient boosting (XGBoost) considering 22 nanoparticles descriptors was used to identify the correlation with toxic effects; although the small size of the database prevented the use of more powerful tools, we could identify size *in situ*, shape surface charge and the type of test as the most predictive parameters of toxicity. In this context, understanding the features correlated with nanoparticle cytotoxicity can enhance model interpretation, providing a clear roadmap for predicting the toxicity of nanoparticles (Yu et al., 2021). Simultaneously, extracting knowledge using transparent and explanatory approaches can boost confidence in adopting AI systems across various frameworks. For example, developing quantitative relationships between nanostructure (physicochemical properties) and toxicity offers novel insights into the molecular mechanisms underlying toxicity (Yan et al., 2023). However, much remains to be done to make these automatic approaches more transparent, reliable, understandable, and useable for humans. We can speculate that, based on the data obtained from this type of analysis, AI tools may be applied to the opposite perspective, namely for the purpose of designing and efficiently producing NMs with specific requirements. Indeed, the potential of machine learning in guiding the design process of nanoparticles and contributing to their safe-by-design development has been very recently discussed (Furxhi et al., 2024).

This perspective becomes of even greater value when NMs are designed for biomedical applications, as AI tools may help deciphering the minimal requirements for the development of safe and effective instruments for nanomedicine. Several authors have highlighted the benefit of applying AI methodologies for the design and selection of nanobiomaterials to reduce the experimental costs and allow the rapid translation from bench to bedside (Mendes et al., 2024; Tomitaka et al., 2023). AI has the potential to address key challenges in *in vitro* nanotoxicology and nanomedicine, offering enhanced data analysis, predictive modeling, and experimental optimization. By leveraging AI, researchers can achieve more accurate, efficient, and comprehensive assessments of nanomaterial biocompatibility and target delivery, overcoming the need for *in vivo* studies and greatly reducing the number of *in vitro* experiments, although still producing reliable information on potential nanomaterial behavior.

## Conclusion

The integration of AI in nanotoxicology holds significant promise. AI applications in nanotoxicology can dramatically enhance our ability to predict and understand the toxicological profiles of nanomaterials. Indeed, machine learning algorithms can analyse large datasets to identify patterns and correlations that might be missed by traditional methods. This can lead to more accurate predictions of nanomaterial behaviour, potentially reducing the need for extensive *in vivo* and *in vitro* testing and enabling faster identification of hazardous nanomaterials. Moreover, AI can assist in modelling complex biological interactions, helping to elucidate the mechanisms of nanotoxicity at various biological levels. Nevertheless, several challenges and caveats need to be critically addressed as the field advances, such as data quality and availability, as well as protocol standardization for AI models validation and applicability in nanotoxicology.

In conclusion, a systematic application of AI for the development of safe NMs and for the prediction of their interaction with biological systems, especially with a view to their use in nanomedicine, must be stimulated, and above all, it becomes essential to create database repositories that allow access to all the data produced so far, so as to allow a substantial reduction in experimental costs. AI holds great promise for a substantial push for a sustainable development of nanotechnology.

## Data Availability

The original contributions presented in the study are included in the article/supplementary material, further inquiries can be directed to the corresponding author.
